# Vaginal “tunnel flap” technique for urethral neomeatus reconstruction after distal urethrectomy in advanced vulvar cancer: a case report with anatomical insights

**DOI:** 10.3389/fmed.2025.1695521

**Published:** 2025-10-13

**Authors:** Marcello Ceccaroni, Filippo Alberto Ferrari, Giovanni Roviglione, Francesco Bruni, Giulia Mantovani, Giorgio Bogani, Cesare Cristofoli, Gaia Masini, Umberto Leone Roberti Maggiore

**Affiliations:** 1 Department of Obstetrics and Gynecology, Gynecologic Oncology and Minimally-Invasive Pelvic Surgery, International School of Surgical Anatomy (ISSA), IRCCS Sacro Cuore—Don Calabria Hospital, Negrar di Valpolicella, Verona, Italy; 2 Division of Oncologic Gynecology, IRCCS Azienda Ospedaliero-Universitaria di Bologna, Bologna, Italy; 3 Gynecologic Oncology Unit, Fondazione IRCCS Istituto Nazionale dei Tumori, Milan, Italy; 4 Plastic and Reconstructive Surgery Unit, IRCCS Sacro Cuore Don Calabria Hospital, Negrar di Valpolicella, Verona, Italy; 5 General Surgery Department, IRCCS Sacro Cuore Don Calabria Hospital, Negrar di Valpolicella, Verona, Italy

**Keywords:** vulvar cancer, urethral neomeatus reconstruction, vaginal tunnel flap, urethrectomy, surgery

## Abstract

Vulvar cancer with distal urethral involvement requires radical surgery that can severely impact urinary function and quality of life. Urethral neomeatus reconstruction therefore, represents a critical yet underexplored challenge in gynecologic oncology. We report a novel vaginal “tunnel flap” technique designed to recreate a functional urethral outlet after radical vulvectomy and distal urethrectomy. The flap leverages detailed knowledge of the urethrovaginal septum and its vascular supply to create a well-vascularized mucosal tunnel that reproduces native urethral morphology while minimizing suture line tension and the risk of postoperative stenosis. In the presented case report, despite early wound-healing complications, suprapubic urinary diversion enabled complete recovery. Long-term follow-up showed sustained oncologic control, excellent urinary function, preserved sexual activity, and satisfactory aesthetic results. This report highlights the role of precise surgical anatomy in optimizing reconstructive outcomes and proposes a reproducible technique that balances oncologic safety with functional and aesthetic preservation.

## Introduction

Vulvar cancer is a rare malignancy accounting for 2–5% of gynecological cancers and primarily affecting postmenopausal women (1). As outlined in the latest revision of the International Federation of Gynecology and Obstetrics (FIGO) staging, tumor extension beyond stage I may involve adjacent perineal structures, including the lower third of the urethra (2). In these cases, as the mainstay of upfront treatment is surgery, with the aim to achieve adequate surgical margins (1–2 cm), it may be necessary to perform a distal urethrectomy at the time of radical vulvectomy. To restore vulvar form and function and achieve tension-free skin closure, particularly in cases of large defects, various reconstructive procedures have been developed and validated, including skin grafts and local or distant flaps (3). The main goals should be the restoration of an acceptable anatomy respectful of aesthetic symmetry and of micturition, defecation, and sexual functions, whenever possible. Postoperative complications are common due to poor wound healing in the female genital area, which is often compromised by tissue maceration and urinary and fecal contamination. Urethral reconstruction may be part of complex plastic surgery procedures using flaps after extensive vulvectomies with wide anterior defects (4, 5). When distal urethrectomy is performed and vulvar reconstruction does not involve the urethra, the urethral margins obtained after surgical transection are traditionally left unsutured for second-intention healing after fixation of the urethral mucosa to the external sphincter muscle (6). With the aim of improving functional outcomes, some oncologic centers have recently published their experience in urethral neomeatus reconstruction using various techniques, demonstrating good results (7, 8). Despite the clinical importance of this issue, literature on urethral neomeatus reconstruction following resection of the distal third of the urethra during radical vulvectomy remains limited. We report a case of urethral “tunnel neomeatus” reconstruction using a vaginal flap after extensive radical vulvectomy with distal urethrectomy for stage II squamous vulvar cancer.

### Case presentation

**Figure 1:**
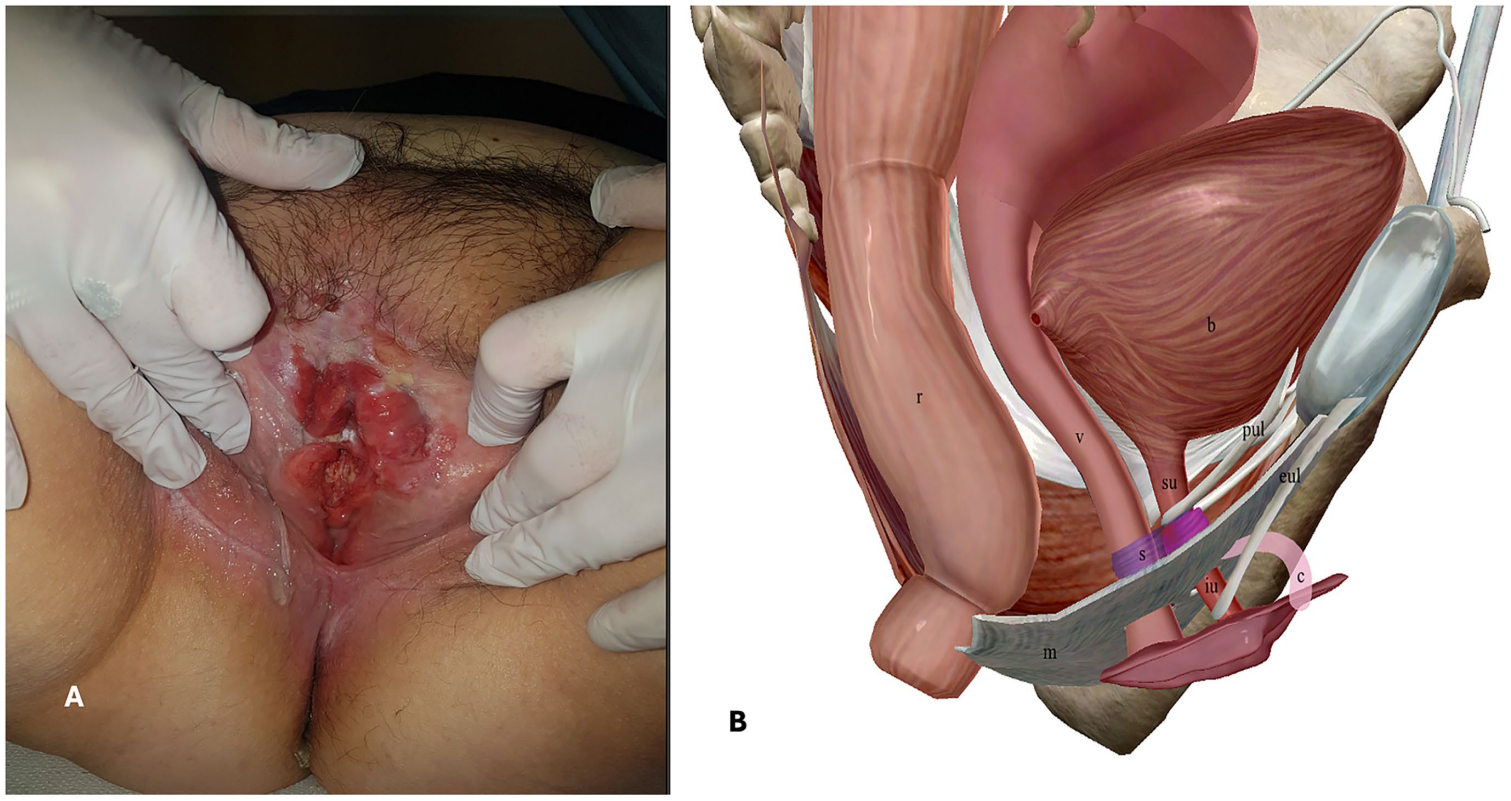
**A**. Pre-operative gynecologic examination. **B**. Anatomy of the female urethra, right-sagittal view. r = rectum, v = vagina, b = bladder, m = perineal membrane, s = urethral sphincter apparatus, su = supra-diaphragmatic urethra, iu = infra-diaphragmatic urethra, c = clitoris, pul = pubo-urethral ligaments, eul = external urethral ligaments.

We present the case of a 65-year-old woman who presented to the Gynecological Department of our hospital in January 2020 with complaints of persistent genital pain and bleeding in the last year. She was overweight (body mass index = 26.17 kg/m^2^) with a medical history of hypothyroidism, insulin-dependent diabetes type 2, scleroderma, and vulvar lichen sclerosus. Gynecologic examination showed an ulcerated vulvar mass of approximately 5 cm involving the cranial third of labia majora and minora, the clitoris, and the caudal third of vagina and urethra (Figure 1A). A biopsy of the vulvar mass performed elsewhere revealed a diagnosis of a well-differentiated keratinizing squamous cell carcinoma of the vulva. Histopathological examination revealed squamous cell nests with prominent keratin pearls, intercellular bridges, and moderate nuclear pleomorphism. There was visible nuclear heterogeneity and scattered atypical mitoses. Immunohistochemical staining demonstrated Ki-67 proliferation index limited to the lower one-third of the epithelial layer, diffuse p16 positivity (block pattern), and PD-L1 expression with a combined positive score (CPS) of >1. High-risk HPV ribosomal acid typing was consistent with HPV 16 positivity.

The patient underwent pre-operative workup comprising 18F-fluorodeoxyglucose positron emission tomography-computed tomography (18F-FDG PET-CT), which did not show suspicious inguinal or pelvic lymphadenopathies, and a transvaginal ultrasonography. After a multidisciplinary consultation, a surgical treatment including radical vulvectomy and bilateral inguino-femoral lymphadenectomy, along with vulvar reconstruction, was planned.

### Anatomical considerations and surgical treatment including neomeatus reconstruction

#### Anatomy of the female urethra

The female urethra is a tubular fibromuscular organ of approximately 41 ± 5 mm length. Its wall is constituted of an epithelial layer (which gradually changes from transitional cells proximally to non-keratinized stratified squamous cells distally), a submucosal layer, an inner circular and outer longitudinal muscular layer and an adventitial layer, also called urethral fascia. The female urethra crosses the urogenital diaphragm and therefore can be conceptually divided into three parts: (1) supra-diaphragmatic urethra; (2) diaphragmatic urethra; and (3) infra-diaphragmatic urethra (9). The urethral fascia continues cranially with the inferior and superior fascia of the urogenital diaphragm and with the vesical fascia. The urethral position is moreover maintained by connective tissue ligaments attached to the pubic bone and by anchoring the supra-diaphragmatic urethra (pubovesical and pubo-urethral ligaments) and the infra-diaphragmatic urethra (external urethral ligaments which are expansions of the suspensory ligament of the clitoris) (10). The urethra is connected to the vagina dorsally by dense connective tissue to form the urethrovaginal septum. The urethral sphincter apparatus is located in the deep perineal space, cranial to the inferior fascia of the urogenital diaphragm (perineal membrane), and surrounds the diaphragmatic urethra. It is responsible for urethral closure at rest and passive continence and consists of two parts: (1) the urethrovaginal sphincter muscle and (2) the urethral compressor muscle (9). Active urethral closure or opening is determined by contraction or relaxation of the pubococcygeus muscle, a hammock-like muscle that stretches from the pubic bone to the coccyx and is part of the *levator ani* muscle. Based on these anatomical considerations, it is evident that the infra-diaphragmatic urethra is excluded from the urethral functional length involved in micturition and urinary continence (Figure 1B).

#### Surgical technique including neomeatus reconstruction

**Figure 2:**
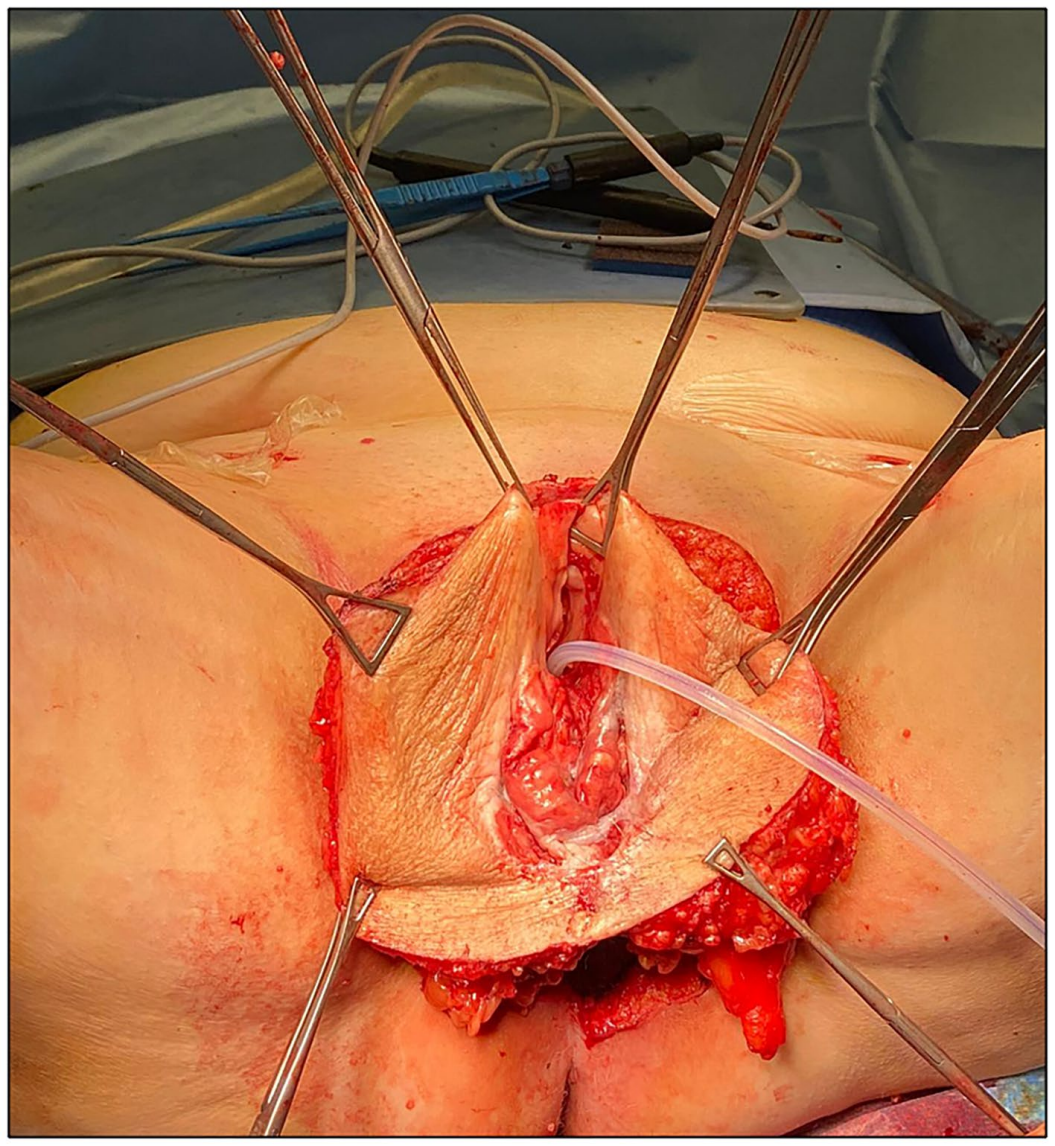
Surgical specimen after complete mobilization and before final medial incision on the vaginal and urethral wall.

The patient received adequate preparation, including trichotomy, bowel preparation, anti-thrombotic prophylaxis, and antibiotic prophylaxis. She was placed in the lithotomy position and underwent general anesthesia. Povidone-iodine solution was used to create an aseptic operative field, a vesical catheter was placed, and the surgical procedure started with systematic bilateral inguino-femoral lymphadenectomy. A dermographic pen was then used to define the surgical incision needed on the vulvar field to obtain adequate disease-free surgical margins (>2 cm). A radical vulvectomy was performed according to the standard procedure, that is, by deepening the dissection to the perineal membrane and dissecting the specimen cranially off the pubic periostium, bilaterally to expose the adductor fascia, and caudally off the perineal body (11). The base of the clitoris was identified, clamped, transected, and ligated. At this time, after complete mobilization of the vulvar specimen bilaterally, cranially, and caudally, a further advancement of the vaginal and urethral wall “en bloc” was obtained by developing the distal third of the rectovaginal septum (with identification and preservation of the *fascia propria recti*) and detaching the distal urethra from the pubic bone by transection of the external urethral ligaments (Figure 2). The final medial incision was then performed with a cold knife on the vaginal and urethral wall at approximately 2 cm from the urethral meatus and vaginal introitus, after vesical catheter removal.

**Figure 3:**
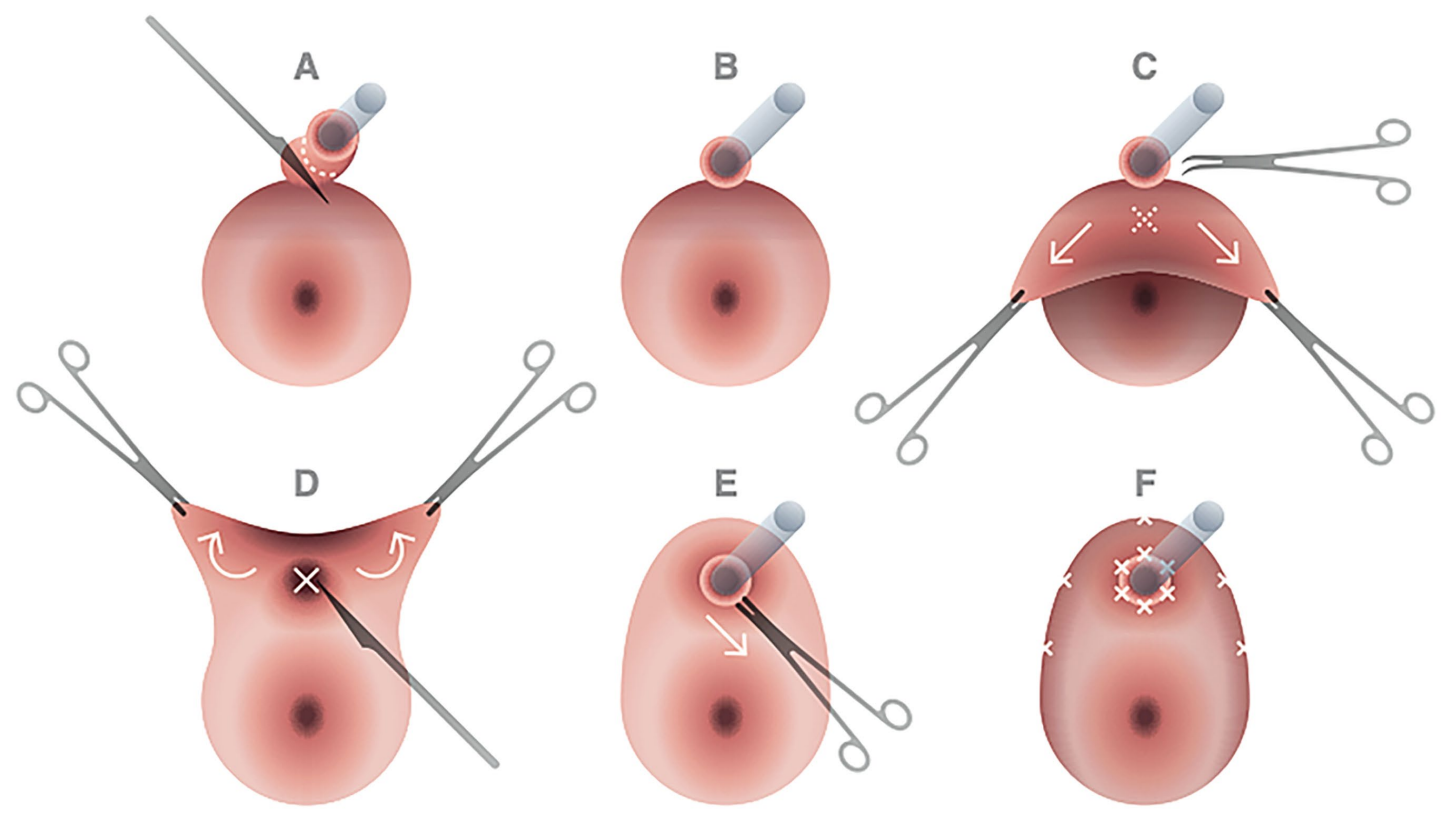
Principal steps of urethral neomeatus reconstruction. After cold knife incision on the urethral wall at approximately 2 cm from the urethral meatus **(A,B)**, the free edges of the ventral vaginal wall were grasped with Allis clamps and tractioned caudally; with Mayo scissors dissection of the urethrovaginal septum connective tissue was performed cranially for approximately 4 cm to obtain an advancement vaginal mucosal flap that could cover without excessive tension the opening of the urethral resected stump **(C)**. Maintaining a gentle traction on the vaginal flap, an X-shaped incision was then performed approximately 1 cm cranially on the vaginal wall to make it correspond to the urethral neomeatus **(D,E)**. A urethral-vaginostomy was then realized with six extroverting single stitches placed in a radial fashion with Vicryl 3-0. The vaginal flap was then anchored peripherally to the skin of the vulvar flaps, creating a tunnel of vaginal mucosa leaning on the course of the re-anastomized urethra **(F)**.

The urethral resected margins were identified by placing Vicryl 3-0 stitches at 6 o’clock and 12 o’clock as a landmark. Vulvar reconstruction was initially realized by the Plastic Surgeon Specialist through V-Y fasciocutaneous advancement flaps. Then the gynecologic team completed the procedure with urethral neomeatus reconstruction, which began with the vaginal “tunnel flap” creation. The free edges of the ventral vaginal wall were grasped with Allis clamps and tractioned caudally. Using Mayo scissors, the urethrovaginal septum connective tissue was dissected cranially for approximately 4 cm to obtain an advancement vaginal mucosal flap that could cover the opening of the urethral resected stump without excessive tension. Maintaining a gentle traction on the vaginal flap, an X-shaped incision was then performed approximately 1 cm cranially on the vaginal wall to make it correspond to the urethral neomeatus. A urethral-vaginostomy was then realized with six extroverting single stitches placed in a radial fashion with Vicryl 3-0. The vaginal flap was then anchored peripherally to the skin of the vulvar flaps, creating a tunnel of vaginal mucosa leaning on the course of the re-anastomized urethra (Figure 3).

### Oncological and functional outcomes

The patient returned to the ward with an 18F silicone Foley catheter inserted into the bladder through the urethra, bilateral inguinal subfascial active tubular drains, and a subcutaneous perineal active tubular drain. She was advised to avoid excessive leg abduction. Wound care with povidone-iodine antiseptic solution and sterile gauze was undertaken twice a day, and laxative use was initially avoided to prevent fecal contamination. Fever developed on the first postoperative day, and unfortunately, the perineal drain was accidentally removed by the patient herself on the second postoperative day; antibiotic therapy with ceftriaxone and metronidazole was initiated promptly after. On the final microbiological reports, *Klebsiella pneumoniae* was found in the urine, and a wound swab culture revealed contamination by *Escherichia coli*, *Klebsiella pneumoniae*, *Enterococcus faecalis*, *Bacteroides fragilis,* and *Bifidobacterium* spp. On the fourth postoperative day, inguinal drains were removed, and an initial wound dehiscence involving the left inguinal wound and the periurethral area (median pubic wound and vaginal attachment to the skin flaps) was noticed. Periodic evaluations by the gynecologic oncologic team and the plastic surgery specialist were carried out, wound dehiscence gradually enlarged and was managed by second-intention healing with the application of hyaluronic acid and collagenase ointment. To avoid urinary leakage and tissue maceration, an epicystostomy was performed on the eighth postoperative day, and the vesical catheter was removed a few days later. The hospital stay lasted 50 days, and the suprapubic catheter was removed after 63 days when complete wound healing was verified and voiding trials were negative.

The final histologic report revealed inguinal lymph nodes free from metastases (0/18), a longitudinal tumor extension of 55 mm, and an infiltration depth of 12 mm; no lymphovascular space invasion was detected, and all the margins were negative and adequately far from the tumor (stage II according to FIGO). The patient started and completed adjuvant radiotherapy on the vulvar and inguinal fields 3 months after surgical treatment.

**Figure 4:**
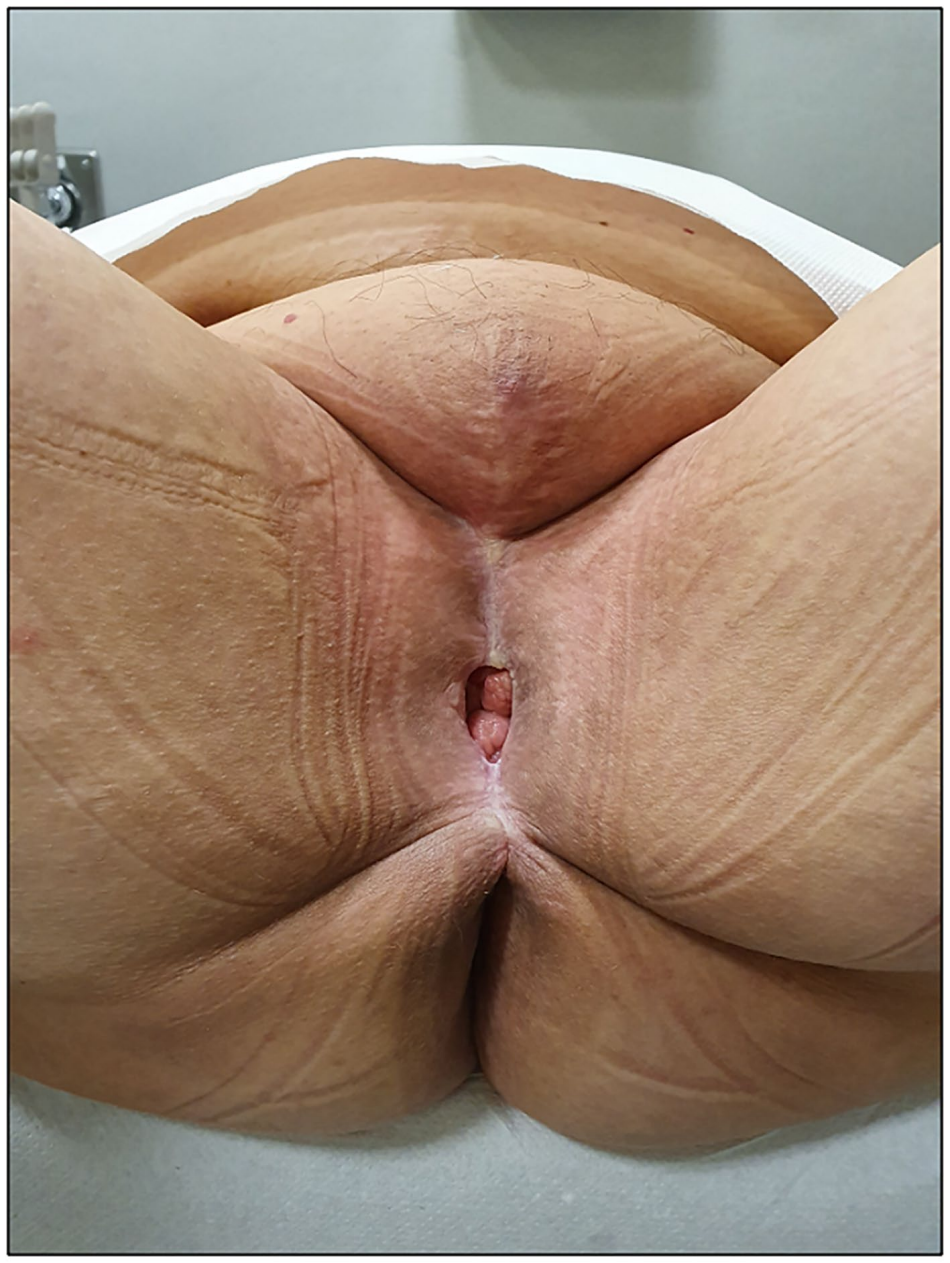
Long-term follow-up (40 months).

After 40 months of follow-up, the patient remained disease-free, with satisfactory aesthetic results—a patent vagina allowing regular sexual intercourse while preserving orgasmic pleasure and normal recto-anal functions, with no fecal incontinence or retention, no signs of obstructed micturition, and no other kind of alterations in urinary functions with an overall good quality of life (Figure 4).

## Discussion

In the field of Gynecologic Oncology, vulvar cancer represents the most frequent malignancy involving the urethra, often requiring complex surgical management, including distal urethral resection and the reconstruction of a neomeatus. The primary surgical challenge lies in balancing oncologic radicality with the preservation of the quality of life, in particular of urinary function, aiming to achieve a durable and functional neomeatus. This balance is particularly difficult to maintain due to the advanced age of many patients, the presence of vulvovaginal atrophy, and multiple co-existing diseases impacting health conditions. Moreover, the frequent need for adjuvant therapies such as pelvic radiotherapy or systemic chemotherapy may further compromise tissue healing and recovery.

Following a demolitive procedure, reconstructing a functional urethral outlet is essential to prevent abnormal wound healing or excessive scarring in the periurethral region, which may determine misdirected urinary stream or urethral stenosis with various degrees of obstructed micturition; If unrecognized and left untreated, these complications may ultimately progress to detrusor dysfunction. In the setting of vulvar cancer surgical treatment, different techniques have been described for the creation of a urethral neomeatus using a vaginal flap. In particular, either an inverted Y-shaped or an X-shaped incision on the dissected vaginal flap may be performed (7, 8). The first technique allows for the formation of two vaginal hemi-flaps that are positioned cranially to encircle the urethral outlet; in the second case, a circular opening is obtained, leaving an intact arched-shape tract of vaginal wall to form the cranial part of the urethral neomeatus (7, 8) (Table 1).

**Table 1:** Comparison of U-shaped and inverted-Y vaginal flap techniques for distal urethral reconstruction.

Surgical details	U-shaped vaginal flap	Inverted ‘Y’ vaginal flap
Skin/mucosal incision	Reverse U-shaped incision on the anterior vaginal wall, apex at the urethral meatus	Longitudinal midline incision of anterior vaginal mucosa with two distal diverging limbs to create an inverted ‘Y’
Flap design	Advancement flap, 2–3 cm in length and 1.5 cm in width, elevated from periurethral vaginal mucosa	Bilateral mucosal flaps mobilized cranially and laterally to reach the periurethral area without tension
Dissection plane	Developed between vaginal mucosa and periurethral fascia, extended proximally toward healthy urethra	Undermining of anterior and lateral vaginal walls for 3–4 cm; meticulous hemostasis before flap advancement
Urethral opening	Ventral urethrotomy at 6 o’clock through the stricture into healthy urethral mucosa	Urethral stump secured to mobilized vaginal flaps, creating a new mucosa-lined meatus
Flap fixation	Apex of flap sutured to apex of urethrotomy; edges sutured to urethral mucosa over 26–28 Fr sound to ensure patency	Flaps sutured to urethral stump with interrupted everting mucosa-to-mucosa sutures (Monocryl 4-0); lateral edges anchored to periosteum or skin edges
Technical limitations	Limited by availability of healthy periurethral tissue; less suitable for very distal urethral loss	Requires adequate vaginal mucosa mobility; technically more demanding due to bilateral dissection

The surgical approach adopted in our case allowed us to obtain negative vulvar., vaginal, and urethral margins, all measuring more than 10 mm from the tumor after fixation. Consequently, the indication for adjuvant radiotherapy was based solely on the extent of tumor infiltration. The preservation of a circular opening on the vaginal flap not only mirrors natural urethral morphology but also minimizes suture line stress and may enhance vascular support to the neomeatus. In consideration of the radial distribution of the tension forces on the flap marginal sutures, the adopted technique of the present case might offer biomechanical advantages compared to traditional U- or Y-shaped flaps, although such hypotheses should be explored in future comparative studies.

Postoperatively, the patient experienced a grade II complication (an infection requiring antibiotic therapy) and a grade III complication (major wound dehiscence >2 cm, necessitating the placement of an epicystostomy catheter) according to the Clavien-Dindo classification system (12). Franchi et al. (8) reported that 13 out of 33 (39.4%) patients undergoing distal urethral resection with neomeatus reconstruction for primary or recurrent vulvar cancer developed a grade II or higher complication. Overall, 11 out of 33 (33.3%) patients experienced a minor or major vulvar dehiscence, all requiring Foley catheterization up to 50 days. In another study, wound dehiscence was the most common complication reported in 11 out of 47 patients (23.4%) and involved the neourethral reconstruction in 2 patients (4.3%). In our case, we opted to promote vulvar wound healing and prevent urinary contamination through the placement of an epicystostomy catheter. Although this may seem an invasive procedure, it proved particularly effective for periurethral wound management. The patient could easily manage the device at home after discharge, and she did not develop urethral stenosis despite Foley catheter removal.

Focusing on postoperative urinary incontinence rate in vulvar cancer patients undergoing partial urethrectomy, data from previous retrospective studies are discordant. In the study by Reid et al. (13), only 4 of the 21 patients with vulvectomy had partial urethrectomy: all four developed either stress or total urinary incontinence (incontinence rate of 100%). In another retrospective case–control study by de Mooij et al. (14), incontinence (urge and stress incontinence together) was reported in 4 out of 18 (22%) patients with a partial urethrectomy compared to 2 out of 17 (12%) patients in the control group (*p* = 0.860). Finally, Hampl et al. (6) attempted to verify the reported symptoms by urodynamic assessment: 5 out of 19 (26%) patients of the study group (urethral resection) complained about urinary disturbances and received urodynamic evaluation. According to urodynamic criteria, 18 out of 19 patients (95%) were classified as continent.

While a worsening or *de novo* presentation of urinary incontinence is described in up to 18% of patients in the cited works on urethral neomeatus reconstruction (7, 8), our patient did not experience urinary incontinence, dribbling, urinary flux deviation, dysuria, or recurrent urinary tract infections. Normal urinary function was confirmed at the 6-month follow-up, after surgery, and at the completion of adjuvant radiotherapy. The main limitation of this report is its single-case design, which precludes generalization of the findings; larger case series and comparative studies are warranted to confirm the reproducibility and long-term functional outcomes of this reconstructive approach.

The present study describes a well-known surgical technique for radical vulvectomy with distal urethrectomy and a novel reconstructive approach to urethral neomeatus reconstruction exploiting anatomical know-how to guide the gynecologic oncologic radical surgeon’s hand. Surgical anatomy provides a different way to approach gynecological malignancies, respecting embryologic heritage and anatomical topography in order to achieve the same radicality while minimizing functional impairment. Given the limited evidence available in the literature, there is not only a significant gap in knowledge, but also a clear need for appropriately trained surgeons with the proficiency to perform complex reconstructive procedures. Expanding surgical expertise in this field is essential to optimize postoperative outcomes, preserve urinary function, and ensure oncologic radicality.

## Data Availability

The raw data supporting the conclusions of this article will be made available by the authors, without undue reservation.
